# Analysis of medicines returned to pharmacies for disposal and estimation of the cost due to medicine wasting

**DOI:** 10.1016/j.rcsop.2022.100133

**Published:** 2022-04-01

**Authors:** Luca Romanelli, Filippo Lucente

**Affiliations:** Department of Physiology and Pharmacology V. Erspamer, University of Rome Sapienza, P.le A. Moro 5, 00185 Rome, Italy

**Keywords:** Disposed medicines, Pharmaceutical waste cost, National health system, Therapeutic class, Remaining dosage units, Remaining validity, NHS, National Health System, NSAIDs, non-steroidal anti-inflammatory drugs

## Abstract

**Background:**

Studies have shown that waste medicines generate a significant cost for the national health system (NHS) in many countries. No data are available on costs and therapeutic classes of unused medicines in Italy.

Objective: Conduct a quantitative and qualitative analysis of unused medicines returned for disposal to selected pharmacies in Rome, Italy, as well as to estimate the related costs for the NHS.

**Methods:**

Medicines returned to 4 pharmacies were collected for 8 months. Therapeutic class, number of remaining dosage units, remaining validity, and reimbursement by the NHS were analysed. The cost of reimbursed medicines was estimated on the prices provided by the Italian regulatory agency (AIFA).

**Results:**

The study sample consisted of 3219 medicine packages containing remaining dosage units, of which 72.4% had expired while 27.6% had not. The average remaining validity of unexpired medicines was 13 months. Medicines reimbursed by the NHS accounted for 73% of the total. Cardiovascular drugs and anti-infectives were the main therapeutic classes (17.2% and 15.2% of total packages, respectively), followed by gastrointestinal drugs, central nervous system (CNS) drugs, non-steroidal anti-inflammatory drugs (NSAIDs), and corticosteroids. The average of remaining dosage units found in the examined packages was 68% of the initial number of units. In terms of cost, antibiotics were the most relevant therapeutic class, followed by cardiovascular drugs, analgesics, corticosteroids, and NSAIDs. The estimated cost for the Italian NHS was € 200,656,780 per year.

**Conclusion:**

Waste medicines constitute a significant, but reducible cost for the NHS. The high prevalence of anti-infectives in the study sample appears to be a distinctive Italian characteristic and may be due to inappropriate prescribing. Policies aimed at reducing waste should improve prescriptive appropriateness and increase the variety of packaging size.

## 1 Introduction

Pharmaceutical expenditure represents a considerable cost to health care systems.^1^ In the last years, the public pharmaceutical expenditure accounted for about 16% of the total expenses of the Italian National Health System (NHS),^2^ whose financial resources have been decreasing in the last years. In the decade between 2010 and 2019, the NHS was defunded by approximately € 37 billion.^3^ This led to staff deficiencies and ultimately had negative consequences on the access to NHS services.^4^ In this context, it is worth looking for ways to save on pharmaceutical costs. Pharmaceutical waste includes expired, unused, spilt, and contaminated pharmaceutical products, drugs, vaccines, and sera that are no longer required and need to be disposed of appropriately.^5^ In Italy (as in other countries), medicines are marketed mostly as pre-packed medications, containing a so-called ‘optimal’ number of posology units. However, it is known that too large, inappropriate pack sizes of medicines still exist.^6^ As a result, a great deal of medicines gets to their expiry date with intact posology units, thus resulting in pharmaceutical waste. Poor adherence to therapy, over-prescription, therapy switching, patient recovery, hospitalization or death etc. all lead to household stockpiling of medicines that are eventually disposed of, and thus wasted.^7^^,^^8^ Pharmaceutical waste may also represent an environmental risk due to improper disposal.^9^^,^^10^

According to Italian law, waste medications must be disposed of by returning them to pharmacies, where they are handed to local waste disposal systems.^11^ There are direct costs linked to the appropriate disposal of waste medicines. In addition, waste is the result of public money spent on prescribed medicines that are eventually not consumed. It is therefore important to analyse waste medicines both quantitatively and qualitatively, to have an estimation of the indirect costs and identify which medicines are more prone to become waste.

Several studies have estimated pharmaceutical waste in different countries, by analysing the medicines returned to pharmacies or other health facilities.^12^, ^13^, ^14^, ^15^, ^16^, ^17^, ^18^, ^19^ Other studies have investigated the prevalence of unused medicines found in households,^20^, ^21^, ^22^ which also contribute to pharmaceutical waste, or found in residual household waste.^23^ All the studies that have also estimated the direct costs resulting from waste medicines found that pharmaceutical waste has significant financial consequences. In the United Kingdom, by extrapolating the data obtained in their study sample to the whole population, Mackridge and colleagues^14^ suggested a value of € 112.5 million wasted annually, but the value may be even higher.^24^ In Spain, the cost estimation in a study performed by Coma and colleagues^15^ was extrapolated to the whole country, thus suggesting a cost of returned medications of € 129.8 million.^24^ In the USA, based on the cost of unused medications found in households, up to USD 117 billion were considered wasted in 2011.^20^ The main therapeutic classes contributing to waste vary from country to country.^7^

As of yet, no known studies have been performed in Italy. The present study quantitatively analysed the returned medicines collected in 4 selected pharmacies in Rome, Italy, for over 8 months. The amount of waste medicines reimbursed by the NHS, and the remaining dosage units and validity were analysed to have a cost estimation of waste medicines. In addition, the study also analysed the sample in terms of therapeutic classes, with the aim of identifying those mainly contributing to the cost due to wasting.

## 2 Methods

The study protocol is provided in Appendix A. The study did not involve human subjects in any way. Hence, according to national law,^25^ and to the Declaration of Helsinki^26^ and international guidelines,^27^^,^^28^ it did not require approval from an ethics committee.

### 2.1 Setting

This was a descriptive study analysing a sample of packed medicines returned to pharmacies by customers for disposal. The sample was collected in 4 community pharmacies located in Rome, Italy.

### 2.2 Selection of pharmacies

**Fig. 1:**
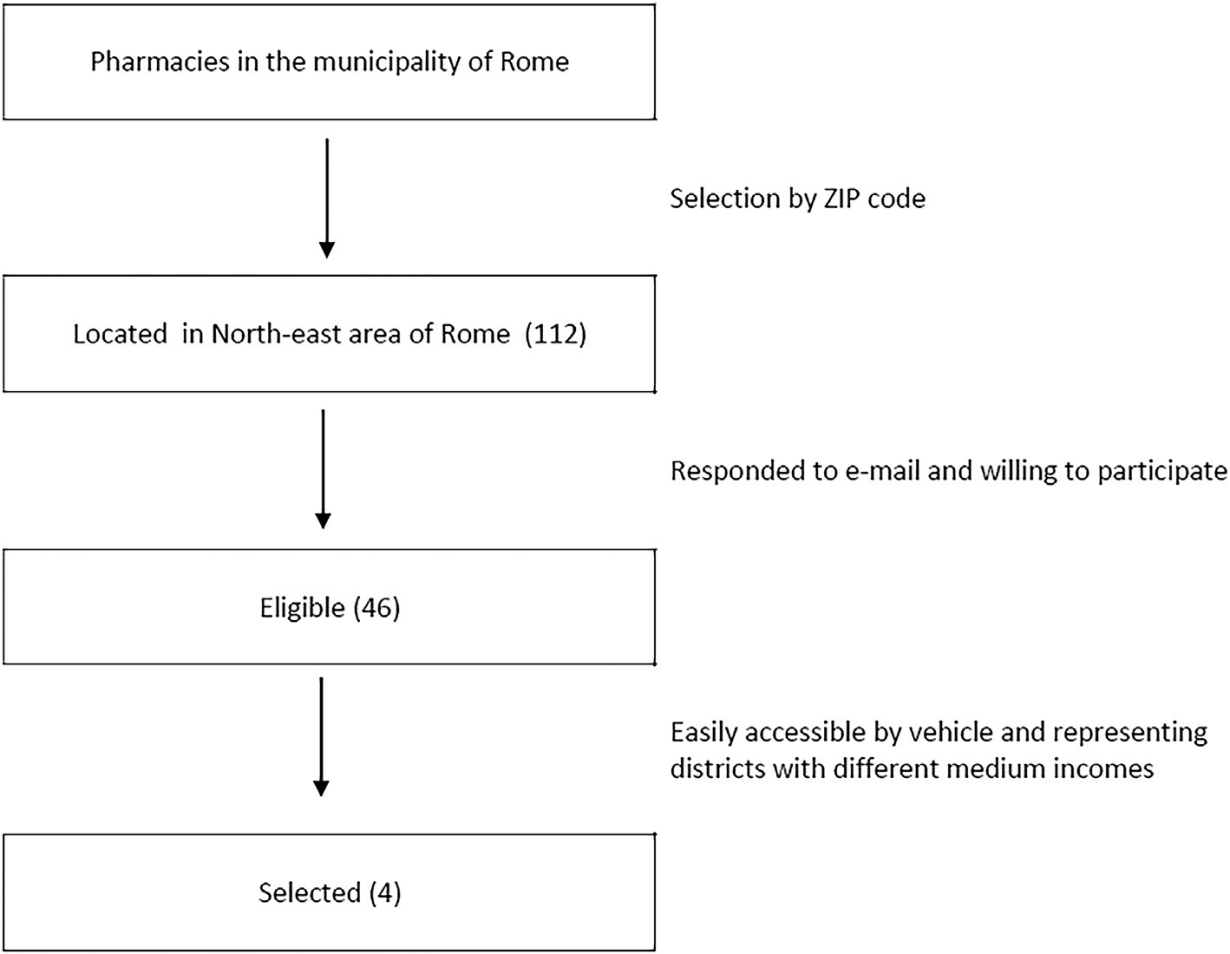
Selection process of the pharmacies that participated in the study.

Fig. 1 shows the flow chart of the selection of pharmacies. They were selected based on 2 criteria: 1) they had to be located in areas representative of the different incomes of the city; 2) they had to be close enough to each other to make the collection of medicines feasible. Eligible pharmacies were all those located in the North-east quadrant of Rome, identified by the ZIP codes, and they were contacted by e-mail. Among those that responded and were willing to participate, 4 pharmacies were selected that represented 4 districts (Municipi II, III, IV and V) with different average incomes based on the data published by the municipality of Rome.^29^ The study was conducted from 1st December 2020 to 31 July 2021.

### 2.3 Collection of returned medicines

The participating pharmacies informed their customers about the collection of expired medicines directly within the pharmacy store (in Italy, expired medicines are generally collected in special containers placed outside pharmacies). The medicine packages were directly delivered by the customers to the pharmacists, who grouped them in collection containers used specifically for the study. At the end of each month, the medicine packages were collected and analysed for each pharmacy involved in the study. For each collected medicine, the following data were recorded: 1) name of the product; 2) active substance(s); 3) the number of remaining dosage units; 4) expiration date. Based on these data, the following additional information was obtained: 5) pharmacological class (according to the 1st or 2nd level of the ATC classification system)^30^; 6) reimbursement by the NHS; 7) remaining validity (in months). The protocol excluded non-quantifiable medicines, i.e. medicines in liquid form for multiple dosing (liquids contained in bottles) and semi-solid forms (gels, creams, ointments etc.) from the analysis. Veterinary drug products, homeopathic products, and food supplements were also excluded. Once analysed, the medicines were regularly disposed of in the appropriate containers outside the pharmacy.

### 2.4 Cost estimation

Cost was only estimated for medicines reimbursed by the NHS. At the end of each month, the cost of the medicines collected in that month was calculated based on the reimbursement prices for the NHS (‘prezzo di riferimento SSN’) provided by the Italian regulatory agency, AIFA.^31^ The price of each medicine package was multiplied by the ratio of the remaining/total dosing units. For medicines that are not reimbursed by the NHS, prices are not fixed and their cost was not estimated.

### 2.5 Data analysis

All data were entered in an Excel® file and descriptive statistics were generated. Statistical significance of the differences among groups was evaluated by ANOVA, using SAS version 9.4 (SAS Institute, Inc., Cary, NC).

## 3 Results

### 3.1 Medicine packages

The study sample consisted of 3219 medicine packages containing remaining dosage units. Among these, 2330 (72.4%) were expired, while 889 (27.6%) were not (i.e. delivered before expire date). The percentage of expired medicines was similar for all therapeutic classes (Table 1). Medicines reimbursed by the NHS accounted for 73% of the total (2352 packages). The number of packages collected monthly from each pharmacy is shown in Appendix B (Table B.1).The absolute number of packages varied between the four pharmacies (ranging from 322 to 1381; median: 758). There were no significant differences between pharmacies in the percentage of reimbursed or expired medicines (Appendix B, Table B.1).

**Table 1:** Analysis of the returned medicine packages according to the therapeutic group. a

Therapeutic group	N of packages	% of total packages	% expired	% remainder dosing units
Cardiovascular system drugs (ATC: C) total	555	17.2	61	67
• *Antihypertensive drugs (ATC C02)*	*448*	*13.9*	*58*	*70*
• *Lipid modifying agents (ATC C10)*	*67*	*2.1*	*76*	*58*
• *Other cardiovascular drugs*	*40*	*1.2*	*68*	*69*
Anti-infectives for systemic use (ATC: J) total	491	15.2	81	77
• *Antibacterials (ATC J01)*	*448*	*13.9*	*82*	*79*
• *Other anti-infectives*	*43*	*1.3*	*78*	*65*
Gastrointestinal drugs (ATC: A02-A07)	361	11.2	65	69
Nervous system drugs (ATC: N) total	352	10.9	64	67
• *Analgesics and antipyretics (non-opioid, ATC: N02B)*	*181*	*5.6*	67	63
• *Antidepressant (ATC: N06A)*	*69*	*2.1*	65	71
• *Others*	*102*	*3.2*	59	73
Anti-inflammatory and antirheumatic products, non-steroids (ATC M01A)	269	8.3	82	69
Glucocorticoids for systemic use (ATC: H02AB)	209	6.5	78	66
Blood and blood forming organ drugs (ATC: B)	187	5.8	62	69
Drugs used in diabetes (ATC: A10)	177	5.5	63	81
Respiratory system drugs (ATC: R)	139	4.3	61	69
Systemic hormonal preparations, excluding sex hormones and insulins (ATC: H)	34	1.1	71	67
Vitamins (ATC: A11)	33	1.0	76	87
Others	412	12.8	75	67
Total	3219	100	72	68

a The second and third level ATC groups are shown in italics.

Cardiovascular drugs and anti-infectives for systemic use were the sample's main therapeutic classes (accounting for 17.2% and 15.2% of total packages, respectively), followed by gastrointestinal drugs, central nervous system (CNS) drugs (mainly non-opioid analgesics), non-steroidal anti-inflammatory drugs (NSAIDs), and corticosteroids (Table 1). Anti-bacterials constituted 91% of the anti-infective group and accounted for 13.9% of the total package sample.

### 3.2 Remaining dosage units

The remaining unit percentage of the total initial units was 68% irrespective of their reimbursement by the NHS. There were no significant differences among therapeutic classes (Table 1).

### 3.3 Remaining validity (shelf-life)

Regarding the 889 packages that had not expired, the mean of the remaining validity was equal to 13 months (range: 1–51 months), with no statistically significant difference between reimbursed (mean: 13.1 months) and not reimbursed (mean: 11.8 months) medicines. There were no significant differences between therapeutic classes.

### 3.4 Cost estimation

Expired and unexpired medicines accounted for 60.9% (€ 16,851) and 39.1% (€ 10,830) of the total cost, respectively. Table 2 shows the estimated cost for medicines reimbursed by the NHS by therapeutic class. In terms of costs, systemic antibacterials were the most relevant therapeutic class, followed by cardiovascular drugs, analgesics, corticosteroids, and NSAIDs.

**Table 2:** Estimated cost by therapeutic group.

Therapeutic group	Cost (Euros)	% of total cost
Cardiovascular system drugs (ATC: C)		
• *Antihypertensive drugs (ATC C02)*	3032	10.9
• *Lipid modifying agents (ATC C10)*	850	3.1
• *Other cardiovascular drugs*	532	1.9
Anti-infectives for systemic use (ATC: J)		
• *Antibacterials (ATC J01)*	4833	17.5
• *Other anti-infectives*	380	1.4
Gastrointestinal drugs (ATC: A02-A07)	1011	3.6
Nervous system drugs		
• *Analgesics and antipyretics (non-opioid, ATC: N02B)*	2147	7.8
• *Antidepressant (ATC: N06A)*	774	2.8
• *Others*	921	3.3
Anti-inflammatory and antirheumatic products, non-steroids (ATC M01A)	1399	5.0
Glucocorticoids for systemic use (ATC: H02AB)	1937	7.0
Blood and blood forming organ drugs (ATC: B)	1382	5.1
Drugs used in diabetes (ATC: A10)	1171	4.2
Respiratory system drugs (ATC: R)	1026	3.7
Systemic hormonal preparations, excluding sex hormones and insulins (ATC: H)	314	1.1
Vitamins (ATC: A11)	311	1.1
Others	5661	20.4
Total	27,681	

To get a rough estimate of the total cost of waste medicines in Italy, the annualized value found in our study (i.e., € 10,380 euro per pharmacy) was multiplied by the total number of pharmacies in Italy (19,331).^32^ The estimated cost was € 200,656,780 per year.

## 4 Discussion

The present study is the first research work regarding medicines returned to community pharmacies in Italy. The number of packages returned varied from pharmacy to pharmacy, which was partly expected based on presumed sales figures. The different involvement of pharmacists and customers may also have contributed to the differences observed. However, the percentage of reimbursed or expired medicines was similar for all pharmacies. During the research period, lockdown measures due to varying intensities of the COVID pandemic alternated with periods of no restriction, but there was no clear correlation between the lockdown measures and the number of medicines returned.

In the sample of medicine packages delivered to pharmacies for disposal, approximately 28% had not yet expired. Similar values for unexpired medications were previously found for medicines returned to pharmacies in Birmingham, UK^14^ and Southern California, USA.^20^ A similar percentage of unexpired medicines was also found in residual household waste in Vienna, Austria (unexpired: 36%).^23^

A rough estimate of the cost of waste medicines reimbursable by the NHS based on a nationwide projection of the data of the present research amounted to approx. € 200 million, which is about 2.6% of the total public pharmaceutical expenditure by the NHS in 2020. The absolute value is in the same range as those found in similar studies in European countries, namely UK^24^, Austria,^23^ Spain,^15^ and Sweden.^33^

The main therapeutic classes accounting for disposed medicines were cardiovascular drugs and systemic anti-infectives, followed by gastrointestinal, CNS, and anti-inflammatory drugs. The abundance of the therapeutic classes in the study sample of waste medicines was not consistent with their incidence on the total number of prescribed medicines in Italy.^34^ It is particularly interesting that the second most abundant waste therapeutic class, i.e. systemic anti-infective drugs (mainly antibacterials), were not included in the 10 therapeutic classes most prescribed in Italy. On the other hand, the two most prescribed therapeutic classes, i.e. drugs for acid-related disorders and statins,^34^ only ranked 7th and 12th as waste medicines. These discrepancies suggest that some therapeutic classes are more prone to be wasted in Italy. The preponderance of cardiovascular drugs as waste or unused medicines is a common finding to almost all previous studies, probably because they are one of the commonly prescribed therapeutic classes for chronic diseases in most countries and frequently associated to changes in therapy and non-adherence.^8^ Focusing on anti-infectives, it is noteworthy that the high prevalence of systemic anti-infectives (ATC J, 15.2%), and particularly antibacterials (ATC J01, 13.9%) found in the present study appears to be a distinctive Italian characteristic because in other European countries the percentage of waste anti-infectives was much lower (4 to 7%). Countries with a percentage of anti-infective waste medicines similar to that found in the present study were Ethiopia,^22^ Egypt,^19^ and Tanzania,^35^ where anti-infective drugs are among the most widely used medicines.^7^^,^^22^ On the other hand, a possible cause of the high prevalence of antibacterials for systemic use may be inappropriate prescribing, which is still a major concern in Italy.^36^, ^37^, ^38^ These data should encourage public policies to improve appropriate prescribing of antibiotics, including the development of practical, evidence-based prescribing guidelines, access to postgraduate training, and better availability of diagnostic tools.^36^^,^^38^

In the case of therapeutic classes used for non-communicable diseases, waste is partly unavoidable, particularly when the patient dies or recovers.^8^ Yet, for therapeutic classes frequently requiring changes in therapy, the availability of packages with a lower number of dosing units and the avoidance of packs that are too large may decrease the amount of waste. Policy-makers could push manufacturers to provide a greater variety of packages,^39^ thus allowing for prescription of shorter supplies. *Re*-evaluation when prescribing refills may also help to decrease pharmaceutical waste while improving adherence.^39^, ^40^, ^41^, 42.

### 4.1 Limitations

This study has limitations to consider. As to the estimation of costs, it was assumed that the pharmacies included in the study are representative of all Italian pharmacies with regard to medicine usage and disposal, but it is not sure to what extent they actually are. The 4 selected pharmacies are located in districts with different average incomes to ensure a good representativeness of the sample. However, the region to which Rome belongs, Lazio, had a pharmaceutical gross expenditure per capita 11.7% higher than the national mean in 2020,^2^ and this may be a source of overestimation. On the other hand, a sure source of underestimation was the exclusion from collection and analysis of non-quantifiable medicines, i.e. medicines in liquid form for multiple dosing (liquids contained in bottles) and semi-solid forms (gels, creams, ointments etc.). An additional factor potentially leading to underestimation is the improper disposal of medicines (i.e., in the trash, sink, toilet, or giving them to a friend or a relative).^40^ The study did not estimate the value of medicines not reimbursed by the NHS, for which the pricing is free (not state-regulated as for reimbursed medicines), and prices vary across pharmacies. However, the expense for unreimbursed medicines contributes to the total expense and the State indirectly pays for it because the cost of drugs is partially tax deductible.

## 5 Conclusion

This study indicates that medicine waste constitutes a significant, yet reducible cost for the NHS. This finding should stimulate the adoption of policies aimed at reducing waste. Cardiovascular drugs and systemic antibacterials were the main therapeutic classes found in the waste sample and the most relevant classes in terms of cost. The high prevalence of systemic antibacterials may be linked to inappropriate prescribing of these drugs in Italy, which, unfortunately, is a widespread practice throughout the country. This should further encourage public policies to improve appropriate prescribing. For therapeutic classes other than antibacterials, pharmaceutical waste may be reduced by prescribing them in shorter supply, which could be facilitated by making a greater variety of drug packages available.

## Funding

This research did not receive any specific grants from funding agencies in the public, commercial, or non-profit sectors.

## Declaration of Competing Interest

None.

## Appendix A Supplementary data

Supplementary material 1 Image 1

Supplementary material 2 Image 2
