# Supplementary material for: Analysis of medicines returned to pharmacies for disposal and estimation of the cost due to medicine wasting
Source: Explor Res Clin Soc Pharm. 2022 Apr 1;5:100133. doi: 10.1016/j.rcsop.2022.100133 (PMC9030279; doi:10.1016/j.rcsop.2022.100133)
Supplement: Supplementary file 1 — Supplementary material 1 [file mmc1.pdf]

PROTOCOLLO dello studio "Analisi della composizione e del costo dei medicinali scaduti raccolti nel comune di Roma"

Coordinatore: prof. Luca Romanelli, Dipartimento di Fisiologia e Farmacologia 'Vittori Erspamer', Università di Roma Sapienza

Razionale e scopo. I farmaci scaduti rappresentano uno spreco delle risorse destinate alla salute. Sarebbe utile conoscere le caratteristiche di questo fenomeno al fine di elaborare misure che possano prevenire tale spreco. Con questa indagine si intende quindi effettuare un monitoraggio dei medicinali scaduti nel Comune di Roma, con gli obiettivi di valutare l'impatto economico-sanitario ed individuare i medicinali a maggiore rischio di spreco.

Metodi dello studio.

Farmacie. Saranno coinvolte nello studio 4 farmacie, tutte localizzate nel quadrante nord-est del Comune di Roma, selezionate in modo da rappresentare zone con diverso reddito.

Raccolta dei dati. La Farmacia che partecipa al progetto comunicherà ai propri clienti che, nel periodo dello studio, i farmaci scaduti da smaltire saranno raccolti all'interno della farmacia, in appositi contenitori che saranno forniti in dotazione dagli organizzatori dello studio. Un componente del gruppo di studio provvederà a prelevare da ciascuna farmacia le medicine raccolte, con cadenza mensile. Saranno quindi raccolti i dati relativi ai medicinali costituiti da unità posologiche distinte (quali compresse, fiale ecc.) escludendo i medicinali il cui residuo non è misurabile (creme, soluzioni ecc.). L'indagine non riguarderà prodotti di integrazione, fitoterapici, veterinari, cosmetici e omeopatici che saranno scartati se

Luca Romanelli, PhD
Università degli Studi di Roma "La Sapienza"
Dipartimento di Fisiologia e Farmacologia 'Vittorio Erspamer'
Tel. (+39) 06 49912575; 3286251060
Mail: luca.romanelli@uniroma1.it

eventualmente presenti tra i medicinali raccolti. I medicinali accumulati saranno poi smaltiti dal gruppo di ricerca, con cadenza mensile.

Analisi dei dati. Per i medicinali così raccolti, saranno determinati: 1) nome del prodotto; 2) principio/i attivo/i; 3) il numero di unità di dosaggio residue; 4) data di scadenza. Sulla base di questi dati saranno poi ottenute le seguenti informazioni aggiuntive: 5) classe farmacologica (secondo il 1° o 2° livello del sistema di classificazione ATC); 6) rimborso da parte del SSN; 7) validità residua (in mesi). Per i medicinali rimborsati dal SSN, il costo sarà determinato mediante consultazione dei prezzi di riferimento SSN pubblicati da AIFA (https://www.aifa.gov.it/liste-di-trasparenza). I dati saranno elaborati mediante Excel®.

Etica. Lo studio non coinvolge in alcun modo soggetti umani né ha come oggetto la tutela della salute. In accordo con la legge italiana (DM 08/02/2013), la dichiarazione di Helsinki e le Linee Guida internazionali, non è quindi richiesta l'autorizzazione da parte di un comitato etico.

Roma, 1 settembre 2020

Firma del Coordinatore (prof. Luca Romanelli)

Luca Romanelli, PhD
Università degli Studi di Roma "La Sapienza"
Dipartimento di Fisiologia e Farmacologia 'Vittorio Erspamer'
Tel. (+39) 06 49912575; 3286251060
Mail: luca.romanelli@uniroma1.it
