# Supplementary material for: Analysis of medicines returned to pharmacies for disposal and estimation of the cost due to medicine wasting
Source: Explor Res Clin Soc Pharm. 2022 Apr 1;5:100133. doi: 10.1016/j.rcsop.2022.100133 (PMC9030279; doi:10.1016/j.rcsop.2022.100133)
Supplement: Supplementary file 2 — Supplementary material 2 [file mmc2.docx]

**Table B.1.** Analysis of medicines returned to the selected pharmacies during the research period (December 2020 – July 2021)

|  |  |  |  |  | MOnth |  |  |  |  |  |  |  |
| --- | --- | --- | --- | --- | --- | --- | --- | --- | --- | --- | --- | --- |
| Pharmacy |  | December  2020 | January  2021 | February  2021 | March  2021 | April  2021 | May  2021 | June  2021 | July  2021 | total per pharmacy | % OF MEDICINES REIMBURSED BY nhS | % of expired medicines |
|  | 1 | 26 | 34 | 55 | 62 | 37 | 43 | 52 | 52 | 361 | 68% | 75% |
|  | 2 | 256 | 254 | 118 | 157 | 100 | 128 | 183 | 185 | 1,381 | 69% | 73% |
|  | 3 | 211 | 124 | 82 | 179 | 51 | 77 | 40 | 391 | 1,155 | 76% | 71% |
|  | 4 | 27 | 31 | 34 | 34 | 44 | 49 | 44 | 59 | 322 | 68% | 68% |
|  | *Total per month* | *520* | *443* | *289* | *432* | *232* | *297* | *316* | *687* |  |  |  |
